# BayesPI-BAR: a new biophysical model for characterization of regulatory sequence variations

**DOI:** 10.1093/nar/gkv733

**Published:** 2015-07-21

**Authors:** Junbai Wang, Kirill Batmanov

**Affiliations:** Pathology Department, Oslo University Hospital—Norwegian Radium Hospital, Montebello 0310, Oslo, Norway

## Abstract

Sequence variations in regulatory DNA regions are known to cause functionally important consequences for gene expression. DNA sequence variations may have an essential role in determining phenotypes and may be linked to disease; however, their identification through analysis of massive genome-wide sequencing data is a great challenge. In this work, a new computational pipeline, a Bayesian method for protein–DNA interaction with binding affinity ranking (BayesPI-BAR), is proposed for quantifying the effect of sequence variations on protein binding. BayesPI-BAR uses biophysical modeling of protein–DNA interactions to predict single nucleotide polymorphisms (SNPs) that cause significant changes in the binding affinity of a regulatory region for transcription factors (TFs). The method includes two new parameters (TF chemical potentials or protein concentrations and direct TF binding targets) that are neglected by previous methods. The new method is verified on 67 known human regulatory SNPs, of which 47 (70%) have predicted true TFs ranked in the top 10. Importantly, the performance of BayesPI-BAR, which uses principal component analysis to integrate multiple predictions from various TF chemical potentials, is found to be better than that of existing programs, such as sTRAP and is-rSNP, when evaluated on the same SNPs. BayesPI-BAR is a publicly available tool and is able to carry out parallelized computation, which helps to investigate a large number of TFs or SNPs and to detect disease-associated regulatory sequence variations in the sea of genome-wide noncoding regions.

## INTRODUCTION

The rapid development of technology has made it increasingly easier to characterize genetic variants in humans and many other species (1). In most species, the number of DNA sequence variants between individuals range from thousands to millions, and some of these sequence variations are linked to traits and diseases. A good understanding of sequence variations in gene regulation may not only reveal disease-associated sequence variants and gene functions but also improve future diagnoses and therapies. Advances in high-throughput sequencing techniques have allowed researchers to carry out genome-wide genetic and epigenetic studies in human disease. In human cancer research, thousands of tumor and normal samples have been sequenced by international consortia such as The Cancer Genome Atlas (TCGA) and the International Cancer Genome Consortium (ICGC) (2,3). The analysis and interpretation of these massive sequence data sets present a great challenge.

The characterization of mutations poses different challenges within and outside gene coding regions. Generally, many useful gene-based features for sequence variations occur within coding regions because of their immediate effect on the encoded amino acid sequence, splicing, protein function and structure (4). Several state-of-the-art computational tools have been developed to predict whether or not a given coding variation may affect the gene function (5–7). However, for the noncoding part of variations, the identification of functional driver mutations, such as disease-associated sequence variants, is hindered by lack of data (8–10). Nevertheless, noncoding variations, such as a single nucleotide polymorphism (SNP), are known to possibly affect gene regulation by either disrupting existing transcription factor (TF) binding sites or creating new ones (11). In this way, an SNP may destroy a link in the gene regulatory network or create a new link, thereby activating or inhibiting target gene expression. Many cases of such regulatory mutations have been studied, for example, the mutation (dbSNP ID rs281864518) in the promoter of the *HBB* gene next to the CCAAT motif, which affects the binding of C/EBP TF causing β+ thalassemia (12); the mutation (HGMD ID CR062116) in a nongenic region between the *α-globin* genes and their upstream regulatory elements, which creates a new binding site for GATA1 TF, leading to α thalassemia (11); and the mutation (dbSNP ID rs689466) upstream of the *PTGS2* gene, which creates a c-MYB binding site and increases the risk of esophageal cancer (13). Thus, establishing connections between functional noncoding mutations and TF binding is an important task. Typically, one has to guess, or determine experimentally, which TF binding affinity is affected by the mutation. When the functional role of the sequence mutation is uncertain, such as in somatic mutations in tumor samples, the problem becomes even more difficult because it is not known which sequence variants may affect the TF binding. In addition, there are at least hundreds of thousands of putative mutations in an individual genome. Doing extensive experimental tests on every mutation, most of which are neutral (14), is not practical. Therefore, completing the task requires robust computational methods that are able to identify significant changes in TF binding affinity due to sequence variations.

For many human TFs, the data about their binding strength to particular DNA sequences are available in the form of position weight matrices (PWMs) (15). A PWM describes how likely the protein is to be bound to a particular sequence, considering every nucleotide independently. Generally, this information alone is not enough to determine whether or not a given mutation will significantly affect the TF binding. For example, the scanning of PWMs on DNA sequences showed a poor performance compared with the sequence conservation scores (16). Advanced mathematical models, such as sTRAP (17) and is-rSNP (18), have been designed to address this problem. Nevertheless, these earlier methods do not consider two important things: the chemical potential (or protein concentration; the number of protein molecules in the nucleus) and the direct protein–DNA interaction. In the present work, a new Bayesian method for protein–DNA interaction with binding affinity Ranking (BayesPI-BAR) is proposed, which includes information on both chemical potentials and putative direct protein–DNA interactions. Because the protein concentration is not known in BayesPI-BAR, a plausible range for this parameter is estimated from ENCODE ChIP-seq experiments, and then a principal component method is used to combine the predictions from multiple chemical potentials.

The goal of BayesPI-BAR is similar to that of sTRAP: given a known functional mutation in a noncoding region, find which TF binding it affects the most. Here, only single nucleotide polymorphisms (SNPs) are considered. Specifically, given an SNP and a list of PWMs of known TFs, the method sorts the TFs by decreasing significance of the binding affinity changes caused by the SNP. Two such sorted lists are produced: one for positive change, meaning that the binding affinity is increased by the SNP (creating a binding motif), and the other for negative change (disrupting an existing binding motif). BayesPI-BAR has been validated on a set of 67 regulatory SNPs that are known to be functional and the affected TFs have been identified experimentally (16,19) by using 2065 PWMs collected for 617 unique human TFs in (15). Of these 67 SNPs, 47 (70%) had the true TFs ranked in the top 10 by BayesPI-BAR. In the future, this new method may become an integrated part of a more advanced computational tool, such as FunSeq2 (20), CADD (21) and GWAVA (22), for annotating functional noncoding regulatory variants in disease.

## MATERIALS AND METHODS

### Human ChIP-seq data

In this work, human ChIP-seq TF binding data (i.e. YY1, IRF1, USF1, SP1, NFKb, AP2, GATA, HNF-4 and CEBP) at various cell lines were obtained from ENCODE (23). The called peaks in ChIP-seq experiments were downloaded from the UCSC genome browser. We used peaks from the ENCODE uniform peak calling pipeline. To estimate the TF chemical potentials in various cell lines, 200 bp DNA sequences centered at the point source of each called peak were extracted from the human genome hg19 reference sequence. The overall enrichment in the peak region, which is computed in the uniform pipeline (23), was used as the signal for the estimation procedure.

### Human regulatory mutations

Two sets of regulatory mutations were collected, in which the TF binding is known to be affected by the sequence variations. The first set contains 20 regulatory SNPs (16) that are known to reduce or enhance the TF binding, although few of them are associated with disease. The second regulatory mutation data set includes 14 SNPs from (19), 4 SNPs from (10) and 29 SNPs found in the HGMD database (24). In this data set, the regulatory mutations not only affect the TF binding but are also associated with human disease. The complete description of all 67 mutations is available in the supplementary Excel file.

### Human position-specific weight matrices

To compute the *in silico* TF binding affinity at the DNA sequence, 2065 PWMs representing about 617 unique human TFs were downloaded from an earlier publication (15). This resource includes the collections from TRANSFAC (25), JASPAR (26), ENCODE (23), protein binding arrays (PBM) (27) and high-throughput SELEX (HT-SELEX) (28) for human TFs.

### Rare and common variants from 1000 genomes

We obtained genotype VCF files of the 1000 Genomes Project (29) from the public FTP site ftp://ftp.1000genomes.ebi.ac.uk/vol1/ftp/release/20110521/, which contains ∼38 million SNPs for 1092 human genomes. There are four major ethnic groups in the 1000 Genomes Project (i.e. European, Asian, African and American). The SNP calling was based on both high-coverage (80x) exome and low-coverage (5x) whole genome sequencing. In this study, we considered only SNPs located ±500 bp to the transcription start site (TSS) of protein coding genes, and the gene annotation was based on GENCODE7 (30). Rare (∼141914) and common (∼80561) variants of the 1000 genomes were obtained by applying VCFtools (31) on the downloaded genotype VCF files, with minor allele frequency (MAF) < 0.01 and MAF > 0.01, respectively (32). To assess the difference in TF binding affinity changes between rare and common variants, we applied BayesPI-BAR on randomly selected 0.5 percentages of rare (∼690) and common (∼400) variants from each chromosome thrice. Then we compared the sum of the top 20 positive/negative TFs binding affinity changes of each random selection with the results based on the randomly generated SNPs (∼400) located ±500 bp to the TSS. The significance of the TF binding affinity changes between the rare/common variants and the randomly generated SNPs was evaluated by a Wilcoxon rank-sum test. The *Z*-values of the rank-sum tests are shown in a color-coded heat map.

### Disease-associated regulatory variants from HGMD

We obtained from the HGMD (24) 416 disease-associated regulatory SNPs that are predicted to disrupt TF binding sites. Unlike the previous set of 29 mutations from the HGMD, this set contained mutations with only *in silico* predictions of TF binding site disruptions and no experimental verification. BayesPI-BAR was applied on these SNPs to estimate the TF binding affinity changes. The significance of the binding affinity changes in the 416 SNPs was compared with that of three randomly generated SNPs located ±500 bp to the TSS by using a Wilcoxon rank-sum test. The *Z*-values of the rank-sum tests are shown in a color-coded heatmap.

### Sequence conservation scores

The sequence conservation score was obtained from a previous publication on GERP++ (33). The score was computed by aligning the reference genomes of human and 33 other mammalian species and then counting the differences on a per-nucleotide level. For each base pair of the human genome with enough multiple species coverage, a rejected substitution (RS) score was provided. The RS scores were either positive (i.e. indicating a deficit in substitution and therefore evolutionary constraint) or negative (i.e. indicating a surplus in substitutions); these were downloaded from http://mendel.stanford.edu/SidowLab/downloads/gerp/. For each selected mutation, the sequence conservation was assessed by using the RS score at the mutation position.

### Biophysical modeling of protein–DNA interaction

Based on biophysical modeling of protein–DNA interaction (34,35), the Fermi–Dirac form of the protein–DNA binding probability is $P(S) = \frac{1}{{1 + \exp (E \bullet S - \mu )}}$, where *S* represents the sequence to be bound by a protein, *E* is the protein binding energy matrix (PBEM) or position-specific weight matrix (PWM) of a TF, and μ is the chemical potential (or protein concentration; the number of protein molecules per nucleus). In theory, the Fermi–Dirac form of the probability can be approximated by a Maxwell–Boltzmann protein binding function (36), $P(S) \approx \exp ( - E \bullet S)$, when a very low protein concentration is assumed in the calculation. A more detailed description of the biophysical modeling and its applications can be found in previous publications, such as BayesPI (35,37–38), BEEML (39), TRAP (40) and MatrixREDUCE (36).

### Estimation of TF chemical potentials

By using the Fermi–Dirac form of the protein binding probability, if the TF PWM (or PBEM) and the TF binding sequences (i.e. 200 bp DNA sequences centered at ChIP-seq called peaks) are known, then the chemical potential of the TF in a particular *in vivo* protein–DNA interaction experiment can be estimated by a Bayesian nonlinear regression model. In the regression model, the measured TF occupancy data (i.e. ChIP-seq tag counts) represent the response variable, and the predicted protein binding probability (affinity) is the explanatory variable with known PWMs but unknown chemical potentials. This calculation is applied in a new version of BayesPI2+ (37), in which the TF chemical potentials at various conditions or cell lines are estimated by fitting a known PWM to an *in vivo* ChIP-seq experiment. In the present work, the data sets used to estimate the chemical potentials are not the same as those used to compute the protein binding affinity by BayesPI-BAR. The former considers the DNA sequences around the ChIP-seq called peaks and the PWMs from the JASPAR database, whereas the latter uses the flanking region sequences of regulatory SNPs and the PWMs from a recent publication (15). Finally, BayesPI-BAR needs only a dynamical range of chemical potentials, which can be either defined manually or estimated from *in vivo* TF binding data, to obtain the TF ranking order by integrating predictions from multiple chemical potentials.

### Ranking the effect of sequence variation on TF binding by shifted differential binding affinity

The *in silico* calculation of the TF binding affinity on DNA sequences by applying the biophysical model has long been used (40,41). Recently, the differential binding affinity (dbA) was introduced to distinguish between direct and indirect protein–DNA interactions (42):

\begin{equation*} {\rm{dbA}}(S_i ) = w\sum\limits_{l = 1}^{N - m + 1} {P_{i,l} \left( {S_i } \right)} - \frac{{\sum\limits_{r = 1}^R {w\sum\limits_{l = 1}^{N - m + 1} {P_{i,l,r} \left( {S_{i,r} } \right)} } }}{R}, \end{equation*}

where $S_i$ and $S_{i,r}$ represent a DNA sequence and its randomly mutated variant, respectively; *N* is the length of the sequence, *m* is the length of the PWM, *w* is a weight coefficient, *R* is the total number of random shuffling of DNA sequence *S_i_*, and $P_{i,l} \left( {S_i } \right)$ and $P_{i,l,r} \left( {S_{i,r} } \right)$ represent the estimated protein–DNA binding probability at sequence *S_i_* and at the randomly mutated sequence $S_{i,r}$, respectively. For the protein–DNA binding probability, either a Fermi–Dirac (with chemical potentials) or a Maxwell–Boltzmann (without chemical potentials) form of the protein–DNA binding probability can be used in the calculation. In addition, an expected *P*-value for direct TF binding to a DNA sequence *S_i_* is provided $P\left( {E_i } \right) = \frac{{\# of\sum\limits_{l = 1}^{N - m + 1} {P_{i,l} \left( {S_i } \right)} >\sum\limits_{l = 1}^{N - m + 1} {P_{i,l,r} \left( {S_{i,r} } \right)} }}{R}$. The *P*-value is used to filter putative indirect TF–DNA interactions when computing the TF binding affinity to a DNA sequence for a set of collected TF PWMs. In the new BayesPI-BAR program, the chemical potential μ can be manually adjusted for a given PWM (in the present work, μ ranges from 0 to −23) in the above-mentioned calculations.

After removing the putative indirect TF–DNA interactions from a set of PWMs (i.e. the 2065 collected PWMs), the difference in TF binding affinity between the reference ($S_{i,reference}$) and the mutated ($S_{i,mutated}$) DNA sequence is quantified by a new shifted differential binding affinity (δdbA), where $\delta dbA_i = dbA(S_{i,reference} ) - dbA(S_{i,mutated} )$. For a given sequence variation, if all available δdbA values of the putative direct TF–DNA interactions (i.e. $P\left( {E_i } \right) < 0.05$) are sorted, then the TFs with the strongest binding effect by the sequence variation can be identified. Here, the positive and the negative δdbA values are sorted separately (i.e. both the largest positive and the smallest negative δdbA are ranked as one).

### Combining the shifted differential binding affinities from multiple chemical potentials

An exact chemical potential for a given TF under a specific condition or cell line is usually not known. BayesPI-BAR uses a range of chemical potentials to estimate the TF binding affinity change at a given sequence variation. This results in multiple δdbA values for every PWM. For example, δdbA(μ) varies in scale with changing chemical potentials, and different PWMs may have a different chemical potential that generates the highest δdbA(μ). The task of identifying the ‘best’ chemical potential for each TF, and then comparing the chemical potentials manually, is rather tedious. A new principal component analysis (PCA) method has been developed that combines the predictions from multiple chemical potentials (i.e. μ equals 0, −10, −13, −15, −18 and −20 in the present study) and then automatically finds the TF most affected by a regulatory sequence variation. The hypothesis is that the TF binding most affected by a sequence variation will be the one with the most extreme position in the multidimensional space of δdbA(μ) scores. For a particular PWM, δdbA(μ) scores for six different chemical potentials are generally correlated. The set of δdbA(μ) scores for all available PWMs is distributed along a line in the six-dimensional space (an example of two-dimensional space is shown in Supplementary Figure S1). If all δdbA(μ) scores are projected onto the first principal component axis for the six-dimensional δdbA(μ) space, then the most affected TF will be detected by the newly projected positions.

**Figure 1.**
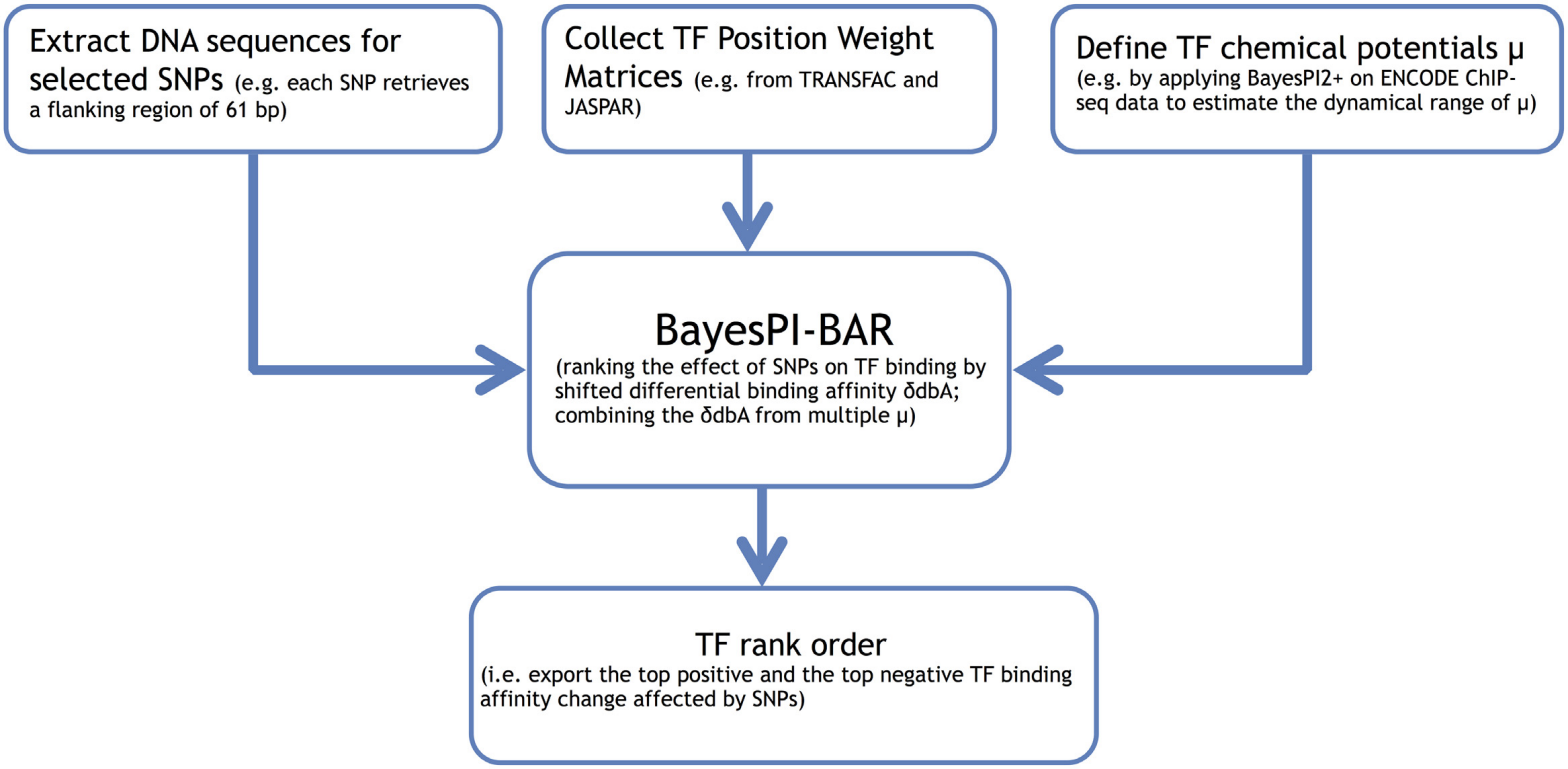
A computational pipeline of the Bayesian method for protein–DNA interaction with binding affinity ranking (BayesPI-BAR). The method is proposed for quantification of the effect of sequence variations on protein binding. BayesPI-BAR uses biophysical modeling of protein–DNA interactions to predict single nucleotide polymorphisms (SNPs) that cause significant changes in the binding affinity of a regulatory region for transcription factors (TFs).

The exact calculation of the principal component method is as follows. Let *D* be the matrix of δdbA(μ) scores, where $D_{i,j} = Sig\left[ {\delta dbA\left( {PWM_i ,u_j } \right)} \right]$ for PWM *i* at chemical potential μ*_j_*, and *Sig* is a sigmoid-like function that normalizes the distribution of the data $Sig\left( x \right) = \log \left( {\left| x \right| + 1} \right) \bullet sign\left( x \right)$; and let *ε* be the eigenvector of the largest eigenvalue for matrix *D^T^D*. Subsequently, the vector $D\varepsilon$gives the projected scores of all PWMs to the first principal component axis; these are the integrated δdbA(μ) scores for predictions based on multiple chemical potentials. BayesPI-BAR will use the projected new scores in the final TF ranking. The scores computed by the PCA procedure are determined up to a multiplicative factor. The sign of the mean δdbA across all chemical potentials is used to recover the positive and negative sides of the ranking. Figure 1 shows the complete pipeline of the BayesPI-BAR framework.

### TF ranking accuracy evaluation

To assess the prediction accuracy of various programs, we first used a cumulative accuracy plot to show the performance BayesPI-BAR, sTRAP and is-rSNP. In this plot, the *X*-axis represents the predicted rank, and the *Y*-axis shows the fraction of mutations for which the true TF appears with the corresponding or a lower rank. Then scatter plots are used to compare the predicted ranking of all mutations by BayesPI-BAR with that by sTRAP/is-rSNP. In the scatter plots, each mutation appears as a circle, with the *X*-axis denoting the ranking of true TFs given by BayesPI-BAR, and the *Y*-axis indicting the rank provided by sTRAP/is-rSNP. Because lower ranks mean better predictions, all mutations above the *X* = *Y* line will be ranked better by BayesPI-BAR, and those below the line will be ranked better by sTRAP/is-rSNP. The significance of the ranking difference in a scatter plot is evaluated by a Wilcoxon signed-rank test.

### Distinguishing verified mutations from randomly generated regulatory variants

In this work, the collected 67 verified mutations that affect TF binding sites are assumed to be functional mutations and are combined with three sets of randomly generated regulatory variants (∼300 SNPs; ±500 bp to the TSS). Three computer programs (BayesPI-BAR, CADD (21) and FunSeq2 (20)) are used to calculate a putative functional mutation score for each variant, respectively. For BayesPI-BAR, the mean absolute δdbA value of the top 20 predicted TFs in both positive and negative directions is used as a prediction score. For CADD and FunSeq2, the default parameter settings are used to obtain the corresponding score for each mutation. The significance of the score difference between the verified and the randomly generated variants is evaluated by a Wilcoxon rank-sum test. Because a higher score indicates that the mutation is more likely to be functional and to affect the phenotype, we expect that the set of scores given by a program to the verified mutations will have a higher median value than that given to random mutations. To evaluate the aforementioned three programs as binary classifiers, we first choose a threshold value for the predicted scores and then divide the set of all mutations into two groups (functional: scores above the threshold, and nonfunctional: scores below the threshold). By considering all possible thresholds for the scores, receiver operating characteristic (ROC) curves can be plotted for the three programs, in which the true positive versus the false positive rates are shown. Usually, the higher the ROC curve, the better the classification accuracy. The area under the curve (AUC) is a numeric measure of the classification accuracy (AUC = 0.5 when a random-guessing classifier is used).

## RESULTS

### Estimated TF chemical potentials in various cell lines

**Figure 2.**
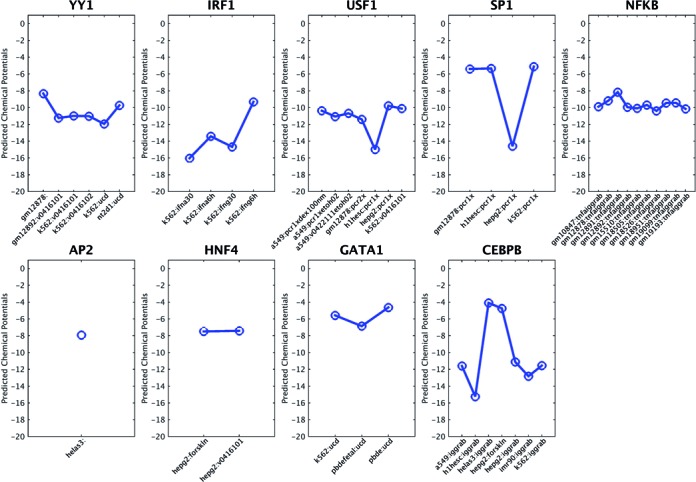
Predicted TF chemical potentials for 9 TFs based on ENCODE ChIP-seq experiments in various cell lines. The known PWMs of 9 TFs were obtained from the JASPAR database. The TF chemical potentials were estimated by fitting the known PWMs and the called ChIP-seq peaks in a Bayesian nonlinear regression model.

For our collected regulatory mutations (*gene*-TF pairs), nine human TFs (YY1, IRF1, USF1, SP1, NFKb, AP2, GATA, HNF-4 and C/EBP) with ChIP-seq experiments are available in the ENCODE project. In total, there are 44 ChIP-seq experiments for the nine TFs in 19 different cell lines. For each TF, the BayesPI2+ program (42) was used to estimate the chemical potentials (protein concentrations) in various cell lines based on the known PWMs from the JASPAR database. The results, as shown in Figure 2, indicate that different TFs prefer different chemical potentials in *in vivo* ChIP-seq experiments. Even for the same TF, the chemical potentials differ in different cell lines. Most of the chemical potentials range between −4 and −18, with a large negative value representing a high protein concentration. This indicates that the TF chemical potential (the number of TF molecules in the nucleus) is an important parameter for biophysical modeling of protein–DNA interaction.

### Ranking TFs based on the shifted differential binding affinity

**Figure 3.**
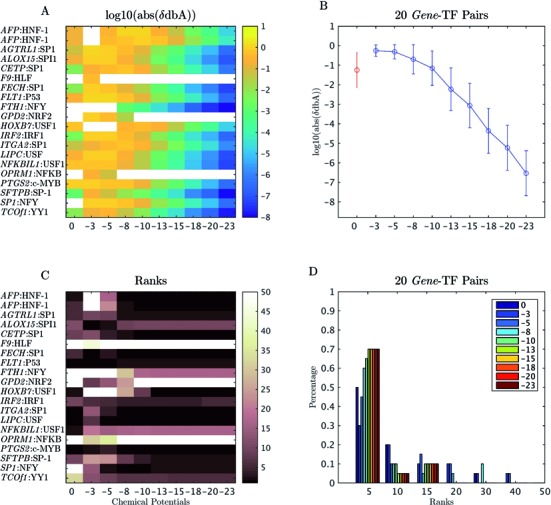
Prediction results of BayesPI-BAR at 20 known regulatory mutations. (**A**) The heat map of log10-transformed absolute δdbA values (the shifted dbA between the reference and the mutated sequence) for 20 known regulatory SNPs (16) that affect the TF binding to the target gene (*gene name*:TF name). The chemical potentials range between 0 and −23 (the large the negative value, the higher the protein concentration or chemical potentials). (**B**) The mean and standard deviation of the log10-transformed absolute δdbA values of 20 known *gene*-TF pairs based on different chemical potentials; the red and blue circles represent predictions based on the Maxwell–Boltzmann function and Fermi–Dirac function, respectively. (**C**) The regulatory mutations of *gene*-TF pairs with the associated TF ranking order, which were predicted for different chemical potentials. (**D**) The bar plot of the percentage of TFs with their associated ranks for different chemical potentials.

**Figure 4.**
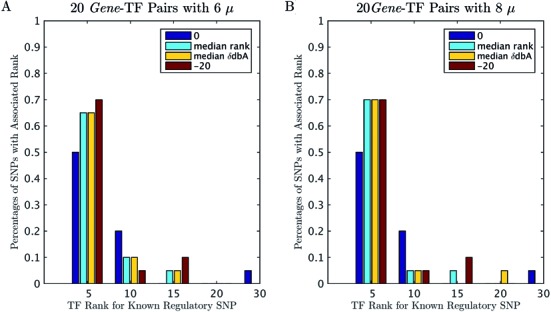
Prediction results of BayesPI-BAR at 20 known regulatory mutations with different chemical potentials. (**A**) Percentages of SNPs with their associated ranks for 20 *gene*-TF pairs; the predicted TF ranks of known regulatory SNPs are based on chemical potential 0, the median ranks or δdbA values for six chemical potentials (0, −10, −13, −15, −18 and −20) and chemical potential −20, respectively. (**B**) The same plot as panel (A) but for eight chemical potentials (0, −5, −8, −10, −13, −15, −18 and −20).

Based on the results shown in Figure 2, a common dynamical range of chemical potentials (i.e. from 0 to −23) was used to compute the shifted differential binding affinities (δdbA) for the 2065 collected human PWMs (15) in a set of 20 regulatory mutations from (16). For each regulatory mutation, 61 bp DNA sequences centered at the mutation change position were extracted from the human genome hg19 reference sequence. The results are presented in Figure 3. In Figure 3A, a heat map of the log10-transformed absolute δdbA values shows that δdbA decreases with increasing TF chemical potential. This is more evident in Figure 3B, in which the mean and the standard deviation of the log10-transformed absolute δdbA values for 20 regulatory mutations across different chemical potentials are plotted. Figure 3C shows a heat map of the predicted ranking order of TFs that are known to be affected by the sequence variations: the darker the color, the higher the predicted TF ranking order; white means that the effect of sequence variation on the TF binding is not recovered by δdbA. The results indicate that, given a very low protein concentration approximation (i.e. the Maxwell–Boltzmann protein binding probability), some of the TF–DNA interactions (e.g. *SP1*:NFY and *GPD2*:NRF2) cannot be predicted. Nevertheless, the predictions based on the Fermi–Dirac form of the function are also poor when extremely low chemical potentials are used in the calculation (i.e. μ = −3), except for *ALOX15*:SPI1. This phenomenon is clearly presented in Figure 3D, in which a bar plot shows the percentages of TFs with their associated ranking for different chemical potentials. The plot shows that ∼50% of the predicted known *gene*-TF pairs have a top ranking of about 5 when the chemical potentials are equal to zero (Maxwell–Boltzmann approximation). Such percentage increased from 30% to 70% when the chemical potentials were increased from −3 to −13 (Fermi–Dirac form of the protein binding probability). Figure 4 shows similar bar plots but for TFs ranked by the median predicted rank (or δdbA) of six and eight chemical potentials (i.e. μ = 0, −5, −8, −10, −13, −15, −18 and −20), respectively. In Figure 4A, the performance of the median ranking based on six chemical potentials is shown to be better than that of the Maxwell–Boltzmann approximation but worse than the best result of Fermi–Dirac form of calculation (i.e. μ = −20). In Figure 4B, the median rank based on eight chemical potentials is close to the best result provided by μ = −20. Similar plots for another 47 verified mutations (10,19,24) are shown in Supplementary Figures S2 and S3, which present similar trends to those in Figures 3 and 4. These results suggest that the chemical potential is protein-specific in the biophysical modeling of TF–DNA interaction; it affects the *in silico* computation of TF binding affinity changes (i.e. δdbA). However, a common dynamical range of chemical potentials can be applied to several TFs because the integration of predictions from multiple chemical potentials will reduce the protein-specific effect of chemical potentials.

### Combining the predictions from multiple chemical potentials

**Figure 5.**
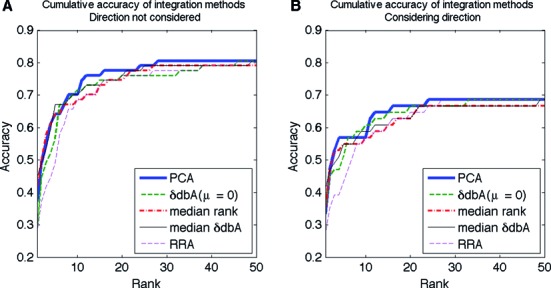
Cumulative accuracy of methods for integrating δdbA scores. **(A)** Cumulative accuracy plots of 67 verified mutations for PCA, median rank, median δdbA and RRA as score integration methods. The accuracy of the ranking based on a single δdbA score (for μ = 0) is provided; the positive/negative direction is not considered. **(B)** The same plots as in panel (A) but also considering the direction of the affinity change.

The results of the previous test suggest that the identification of the impact of sequence variations on protein binding is related to the protein-specific chemical potential in the biophysical modeling of protein–DNA interactions. BayesPI-BAR prefers the integration of predictions from a range of chemical potentials. To evaluate this integrated approach, we applied BayesPI-BAR on 67 verified regulatory SNPs, with the δdbA of every TF computed for six chemical potentials (μ = 0, −10, −13, −15, −18 and −20). The final ranking of the effect of regulatory sequence variations on TF binding is based on a PCA, which combines predictions from multiple chemical potentials. Here, the ranking for positive (creation of new motifs) and negative (destruction of existing motifs) change was done separately. The results are presented in Figure 5 as cumulative accuracy plots, in which the *X*-axis denotes the increasing number of TFs considered in the ranking, and the *Y*-axis represents the cumulative accuracy (i.e. the fraction of mutations for which the true TF appears with the corresponding or a lower rank). In Figure 5A, the direction of the affinity change is not taken into account, which means that we considered the lowest ranking for a true TF with either negative or positive predicted affinity change. The accuracy of four integration methods is shown: PCA, median rank, median δdbA and robust rank aggregation (RRA) (43). Additionally, the results based on the Maxwell–Boltzmann approximation (μ = 0) are also plotted. The figure indicates that the PCA method outperforms the other integration methods by a small margin and is not severely affected by the sequence length (Supplementary Figure S4). Figure 5B shows the results of a similar analysis but with the directions of the predicted affinity changes considered. Here, 51 mutations with known directions of TF binding affinity changes are used, and only the ranking with the correct direction is considered. In this test, all cumulative accuracies are lower than those of the previous test. Nevertheless, the overall trend of performances is the same as that shown in Figure 5A. This suggests that the integration of multiple predictions from different chemical potentials extracts more information than does an individual estimation. The PCA integration method is used in the subsequent data analysis.

### Comparing prediction accuracies between BayesPI-BAR and other binding affinity change ranking methods (sTRAP and is-rSNP)

The performance of BayesPI-BAR was compared with those of other existing methods with similar functionality, such as sTRAP (17) and is-rSNP (18). The sTRAP package was downloaded from its original publication, and the library of PWMs was rebuilt in the local machine, which has the same 2065 PWMs used in the present study. To test the is-rSNP method (18), we used the web service of *in silico* regulatory SNP detection provided by its authors, with the same DNA sequences used in this study. Because is-rSNP does not allow the use of custom PWMs, the TRANSFAC+JASPAR option provided was used in the test. Both tools output a list of PWMs that are predicted to be affected by the given SNP, ordered by decreasing significance. The predicted direction of binding affinity change is also given in the output.

**Figure 6.**
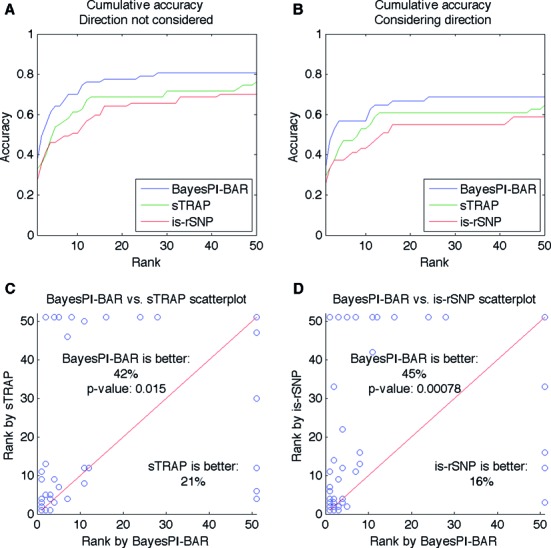
Cumulative accuracy of predicted TF rankings for 67 verified mutations. (**A**) Cumulative accuracy plot for BayesPI-BAR, sTRAP and is-rSNP. The *X*-axis represents the predicted rank, and the *Y*-axis shows the fraction of mutations for which the true TF appears with the corresponding or a lower rank. The positive or negative direction of TF binding affinity changes is not considered. (**B**) Cumulative accuracy plot for BayesPI-BAR, sTRAP and is-rSNP considering the predicted direction of TF binding affinity changes. (**C**) Scatter plot of the predicted ranking of mutations for BayesPI-BAR and sTRAP, with the ranks capped at 51. Mutations above the *X* = *Y* line (red) are ranked better by BayesPI-BAR, and those below the line are ranked better by sTRAP. The *P*-value of the Wilcoxon signed-rank test comparing the paired ranks is given. (**D**) The same plot as in Figure 6C but for BayesPI-BAR versus is-rSNP.

Figure 6A shows a cumulative accuracy plot of the results predicted by the three programs (BayesPI-BAR, sTRAP and is-rSNP) based on the 67 verified mutations, with the *X*-axis denoting the increasing number of TFs, and the *Y*-axis representing the cumulative accuracy. Here, the direction of binding affinity change is not considered in the accuracy calculation. The cumulative accuracy plot reveals that BayesPI-BAR, sTRAP and is-rSNP reach maximum cumulative accuracies of 81%, 76% and 70%, respectively, when the top 50 predicted TFs are considered. When the top 10 predicted TFs considered, the cumulative accuracies of BayesPI-BAR, sTRAP and is-rSNP are 70%, 61% and 51%, respectively. In Figure 6B, the cumulative accuracy of BayesPI-BAR is compared with that of sTRAP/is-rSNP by considering the predicted direction of TF binding affinity changes. All three programs showed lower accuracies in this setup, but BayesPI-BAR still performed better than the other two methods. The decreased accuracies may be mostly due to the exclusion of higher-quality mutation data from Andersen and Epstein sets (16,19), which often do not include information about the direction of the affinity change. In Figure 6C and D, the predicted true TF rankings by BayesPI-BAR are compared with those by sTRAP or is-sSNP in scatter plots. In these tests, the direction of affinity changes is not considered so as to allow the use of more data points. BayesPI-BAR achieved better true TF rankings for 42% and 45% of SNPs, respectively, compared with sTRAP and is-rSNP. On the contrary, only 21% and 16% of SNPs were ranked better by sTRAP and is-rSNP, respectively, compared with BayesPI-BAR. The differences are statistically significant (*P* < 0.02 and *P* < 0.0008 for sTRAP and is-rSNP, respectively). Thus, for most of the 67 verified mutations, BayesPI-BAR provided the same or better prediction accuracy compare with the two other programs.

### Distinguishing verified mutations from randomly generated regulatory variants

**Figure 7.**
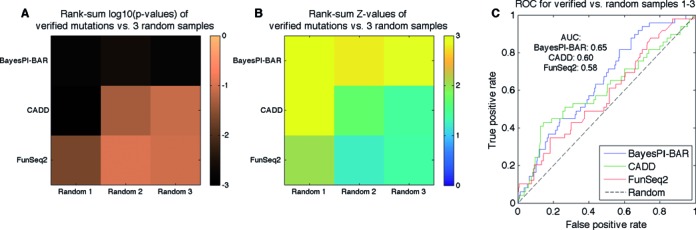
Distinguishing the 67 verified mutations from random ones. The results of three programs (i.e. BayesPI-BAR, CADD and FunSeq2) are compared. (**A**) The log10-transformed *P*-values of the rank-sum tests for the predicted scores of verified mutations versus those of randomly generated ones. Lower *P*-values indicate a more significant difference in predicted scores between the two sets of mutations. (**B**) The *Z*-values of the same tests as in Figure 7A. (**C**) The receiver operating characteristic (ROC) curves for the three programs show the performance of the programs as binary classifiers (i.e. functional versus random mutations) and the area under this curve (AUC) serves as a numeric measure. For a random-guessing classifier (‘Random’), the AUC of the theoretical ROC curve is 0.5.

The putative functional mutation scores between the 67 verified mutations and the three sets of randomly generated regulatory variants, each with ∼120 mutations located at ±500 bp to the TSS of annotated protein coding genes, were compared by Wilcoxon rank-sum tests for BayesPI-BAR, FunSeq2 and CADD. The log10-transformed *P*-values and the corresponding *Z*-values are shown in heat maps in Figure 7A and B, respectively. Because some of the mutations were either removed by FunSeq2 due to internal filtering conditions or have the status of coding mutations in CADD, the results shown in the figure are based only on 49 verified mutations and three sets of randomly generated regulatory variants (each with ∼100 mutations) that are available in both FunSeq2 and CADD as noncoding mutations. The darker the color in Figure 7, the smaller the *P*-value. The heat maps clearly show that the putative functional mutation scores obtained by BayesPI-BAR indicate a strong difference between the 49 verified mutations and the randomly generated variants (median *P* < 0.002). However, the mutation scores provided by either CADD or FunSeq2 show a marginal difference between the verified and the randomly generated mutations (median *P* < 0.05 or *P* < 0.09), with only one of three CADD tests giving a significant *P*-value (*P* < 0.002). The ROC curves for the three predictions are presented in Figure 7C, in which the true positive versus the false positive rate is shown. The AUC values for BayesPI-BAR, CADD and FunSeq2 are ∼0.65, 0.6 and 0.58, respectively. These results suggest that the putative functional mutation scores computed by BayesPI-BAR (considering pure DNA sequence information) are better than those provided by either CADD or FunSeq2 (considering diverse genomic information, including conservation scores) in distinguish functional from nonfunctional mutations.

### TF binding affinity changes in rare and common variants from the 1000 Genomes Project

**Figure 8.**
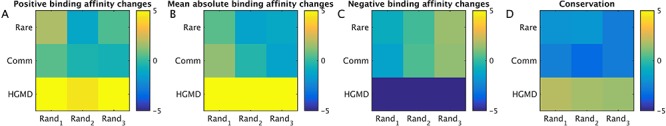
Comparison of TF binding affinity changes between the rare/common/disease-associated variants and the randomly generated ones. ‘Positive binding affinity changes,’ ‘mean absolute binding affinity changes,’ and ‘negative binding affinity changes’ represent the sum of the positive (**A**), the mean absolute (**B**) and the negative (**C**) changes of the top 20 TF δdbA values predicted by BayesPI-BAR, respectively. The above-mentioned three types of binding affinity changes in rare (‘Rare’), common (‘Comm’) and disease-associated variants (‘HGMD’) were compared with those of randomly generated ones (i.e. ‘Rand_1_,’ ‘Rand_2_’ and ‘Rand_3_’) by using Wilcoxon rank-sum tests. ‘Conservation’ (**D**) refers to the comparison of sequences conservations between the real (i.e. rare, common or disease-associated) variants and the randomly generated ones by using Wilcoxon rank-sum tests. All *Z*-values of the rank-sum tests are shown with yellow and blue indicating positive and negative *Z*-values, respectively.

To study the TF binding affinity changes in rare and common variants, we focus on the variants located ±500 bp to the TSS of the annotated protein coding genes (i.e. GENCODE7) because of the low coverage of genome-wide sequencing data in the 1000 Genomes Project (29). First, VCFtools was used to identify rare and common variants from the above-mentioned genomic regions. Then, BayesPI-BAR was applied on a randomly selected subset of rare or common variants (i.e. ∼0.5 percentages of variants from each chromosome), as well as a set of randomly generated regulatory SNPs (∼400) and disease-associated regulatory SNPs (∼416) from HGMD, respectively. The random selection was repeated thrice. In each selection, the sum of the top 20 positive/negative TF binding affinity changes were computed for the rare variants, common variants, disease-associated regulatory SNPs, and randomly generated regulatory SNPs, respectively. The distributions of the predicted positive or negative TF binding affinity changes in the above-mentioned four types of variants are shown in box plots (Supplementary Figure S5). Subsequently, a Wilcoxon rank-sum test was used to evaluate the significance of TF binding affinity changes between the rare/common/disease-associated variants and the randomly generated regulatory SNPs. The *Z*-values of the rank-sum tests are shown in color-coded heat maps (Figure 8A–C), which indicate that both the rare and the common variants have similar TF binding affinity changes when compared with the randomly generated ones. For the rare variants, the median *P*-values of the three tests for positive, negative and mean absolute binding affinity changes are *P* < 0.21, *P* < 0.5 and *P* < 0.3, respectively. For the common variants, the median *P*-values of the same tests are *P* < 0.61, *P* < 0.21 and *P* < 0.2, respectively. However, for the disease-associated variants from HGMD, the TF binding affinity changes are significantly higher than that of the randomly selected one; the median *P*-values of the three tests are *P* < 2.8e-7, *P* < 2.3e-11 and *P* < 1.8e-15 for positive, negative and mean absolute binding affinity changes, respectively.

Additionally, we compared the sequence conservations of the aforementioned four types of variants. The significance of the difference in sequence conservations between the rare/common/disease-associated variants and the randomly generated ones is assessed by a Wilcoxon rank-sum test. The *Z*-values of the tests are shown in a color-coded heat map in Figure 8D. The results indicate that the DNA sequences of both the rare and common variants are much less conserved than that of the randomly generated ones (negative *Z*-values; the median *P*-values of the three tests are *P* < 0.05 and *P* < 0.008 for the rare and common variants, respectively). However, for the disease-associated variants from HGMD, the sequence conservations are slightly higher than that of the randomly generated ones (positive *Z*-values; the median *P*-value of the three tests is *P* < 0.095). These results prompted us to investigate the distribution of various sequence variants around the TSS. The plots are shown in Supplementary Figure S6, which indicates a clear peak of the disease-associated HGMD variants at ±100 bp to the TSS, although the distributions of the rare and common variants are similar to that of the randomly generated one. Nevertheless, there is a small peak of the common variants between −200 bp and +100 bp to the TSS, and a clear drop-off of the rare variants from −100 bp to the TSS.

In summary, by using BayesPI-BAR to compare the TF binding affinity changes among four types of sequence variants (i.e. rare, common, disease-associated and random), we found that the disease-associated variants significantly alter the TF binding affinity changes and that they correlate with a relatively higher conservation score at the mutation position compared with the randomly generated ones. However, both the rare and the common variants have little effect on the TF binding affinity changes and are much less conserved than the randomly generated ones.

## DISCUSSION

A previous work (16) has shown that a simple scan of PWMs on DNA sequence (TF binding affinity) cannot reliably quantify the effect of sequence variation on TF binding compared with predictions based on sequence conservation scores. Thus, several advanced statistical methods such as sTRAP (17) and is-rSNP (18), were developed to address the problem. An important part of the TF binding affinity calculation is determining whether the interaction between a TF and a DNA sequence is significant. In sTRAP (17), an empirically derived statistical model is used to compute such significance scores. A parametric probability distribution is fitted to the set of computed TF affinity scores for each PWM by considering a provided set of background sequences. This allows the computation of a *P*-value for the hypothesis that a given TF PWM binds more strongly to a given sequence than to the background sequence. In is-rSNP (18), a model of the ratio between the reference and the mutated affinity scores is used to test the significance of a mutation to a given TF. In this work, a new biophysical model (BayesPI-BAR) that explicitly considers the difference in binding probability between the background and the given sequence (differential binding affinity (42)) is proposed to address the same problem. This concept was originally developed to distinguish between direct and indirect TF binding, which is closely related to the TF binding affinity changes because functional sequence variations are tightly associated with direct TF–DNA interactions. Additionally, the effect of the chemical potential (or the protein concentration in the cell) on the TF–DNA interaction is also considered in BayesPI-BAR, which provides more information of TF binding variations than does the low protein concentration assumption, such as in sTRAP.

In our study, the importance of chemical potentials in *in vivo* ChIP-seq experiments is presented first in Figure 2, in which the predicted chemical potentials show a clear cell line- and protein-specific behavior. Then, the impact of chemical potentials on the TF binding affinity change is presented in Figure 3 and Supplementary Figure S2, which show decreasing δdbA values with increasing chemical potentials. This is consistent with the theory that many unspecific TF–DNA interactions occur when the number of TF molecules per nucleus is very high and that a high protein concentration results in a minor difference between specific and unspecific TF–DNA interactions (44). Importantly, the significance of TF binding affinity changes, which are influenced by sequence variations, may be blurred when the wrong chemical potentials are used in the protein binding probability. For example, in Figure 3C and Supplementary 2C, the prediction accuracy of BayesPI-BAR is greatly reduced by the use of chemical potentials that are too low (i.e. μ = −3). Therefore, the *in silico* calculation of TF binding affinity changes (i.e. δdbA) through biophysical modeling of TF–DNA interaction is very sensitive to the choice of chemical potentials. A Maxwell–Boltzmann approximation may be insufficient to identify the real effect of sequence variations on TF binding affinity changes. An integration of predictions from multiple chemical potentials (Figure 4 and Supplementary Figure S3) is thus needed.

Motivated by the above results, a new principal component method is designed combining the predicted effect of sequence variations on TF binding from multiple chemical potentials. The results are first compared with the other integration methods, such as the robust rank aggregation (RRA) algorithm and the median rank, as shown in Figure 5, with the highest prediction accuracy obtained by the principal component method. Subsequently, three programs, BayesPI-BAR, sTRAP and is-rSNP, which are designed to predict the effect of SNPs on TF binding, are systematically compared, as shown in Figure 6. Both the cumulative accuracy and the scatter plots indicate that BayesPI-BAR gives significantly better predictions in most of the verified mutations compared with the two other programs (*P* < 0.02 and *P* < 0.0008). Although sTRAP is very similar to the Maxwell–Boltzmann approximation version of BayesPI-BAR, the filtering of putative indirect TF binding targets and the computation of a new δdbA score in BayesPI-BAR greatly improve the prediction accuracy. Generally, the two biophysically originated methods (BayesPI-BAR and sTRAP) surpass the common statistical method (is-rSNP) in identifying TF–DNA interaction affected by sequence mutation changes. However, the lower prediction accuracy of is-rSNP, which was previously reported to be more accurate than sTRAP (18), may partially be explained by the difference in PWMs (i.e. is-rSNP is equipped only with TRANSFAC and JASPAR PWMs). In the present study, no method completely outperforms the others in all 67 verified mutations. In the future, an integration of the results from multiple methods will provide more robust prediction than that achieved by a single method.

In addition, BayesPI-BAR is compared with two other programs (CADD and FunSeq2), which were developed for the identification of functional regulatory mutations by integrating diverse information. Although BayesPI-BAR is not designed for the same purpose, its δdbA (the shifted dbA between the reference and the mutated sequence) may provide *in silico* phenotype information for distinguishing functional (driver) from random (passenger) mutations. This hypothesis is tested; the results are shown in Figure 7, in which the putative functional mutation scores (mean absolute δdbA) provided by BayesPI-BAR show the strongest difference between the 67 verified mutations and the ∼300 randomly generated regulatory variants. In particular, the ROC curves, based on a binary classification of verified and random mutations, suggest that the scores produced by BayesPI-BAR (AUC = ∼0.65) are slightly better than those generated by either CADD (AUC = ∼0.6) or FunSeq2 (AUC = ∼0.58) in distinguishing functional from nonfunctional mutations. This is a very interesting result because the scores computed by BayesPI-BAR only consider the DNA sequence information. In contrast, the two other programs integrate diverse information, such as sequence conservation, disease or gene annotation, TF binding, enhancer-gene linkages and protein interaction network centrality, among others. If our proposed δdbA is integrated into these earlier methods, then a great improvement in functional mutation prediction in the noncoding part of the genome can be expected.

Finally, BayesPI-BAR is used to investigate the TF binding affinity changes among four types of variants: rare and common variations predicted from the 1000 Genomes Project, disease-associated regulatory variants provided by HGMD and randomly selected variations ±500 bp to the TSS. The results shown in Figure 8 suggest that there is no clear difference between the rare and the common variants from the 1000 Genomes Project when their impact on the TF binding affinity changes is considered. In other words, the effect of either rare or common variants on TF–DNA interaction is similar to that of the randomly selected regulatory variants. This may be explained by the low coverage of sequencing depth at non-exome regions in the 1000 Genomes Project, which results in a high error rate and unreliable variant calling (45,46). Second, the heterogeneous population (i.e. European, Asian, African and American) in the 1000 Genomes Project may also weaken the detection of rare sequence variations (32). However, for the disease- associated regulatory variants from HGMD, the predicted TF binding affinity changes by BayesPI-BAR are significantly higher than those of the randomly selected variants (i.e. the median *P*-value of the three tests are *P* < 2.8e-7, *P* < 2.3e-11 and <1.8e-15 for positive, negative and mean absolute binding affinity changes, respectively). Thus, BayesPI-BAR is a very useful tool for detecting sequence variants that disrupt protein binding sites.

In conclusion, the new biophysical model BayesPI-BAR was successfully applied on 67 known regulatory mutations, with a clear improvement shown over other existing computational methods. BayesPI-BAR is a publicly available package (http://folk.uio.no/junbaiw/BayesPI-BAR) that contains C, Perl and R programs. It is capable of parallelized computation under Windows, Linux and Mac OS X operating system, which may significantly reduce the CPU time needed for a large number of PWMs or regulatory mutations. BayesPI-BAR is thus a useful tool for detecting functional driver mutation in the noncoding part of the genome (10) and exploring massive genome-wide sequence data that are constantly generated by large consortia, such as the International Cancer Genome Consortium (3) and the Cancer Genome Atlas (47).

## Supplementary Material

SUPPLEMENTARY DATA
