# Supplementary material for: BayesPI-BAR: a new biophysical model for characterization of regulatory sequence variations
Source: Nucleic Acids Res. 2015 Jul 21;43(21):e147. doi: 10.1093/nar/gkv733 (PMC4666384; doi:10.1093/nar/gkv733)
Supplement: SUPPLEMENTARY DATA [file supp_43_21_e147__index.html]

BayesPI-BAR: a new biophysical model for characterization of regulatory sequence variations — BayesPI-BAR: a new biophysical model for characterization of regulatory sequence variations — SUPPLEMENTARY DATA 

# BayesPI-BAR: a new biophysical model for characterization of regulatory sequence variations

## SUPPLEMENTARY DATA

- SUPPLEMENTARY DATA
- SUPPLEMENTARY DATA
