# Supplementary material for: BayesPI-BAR: a new biophysical model for characterization of regulatory sequence variations
Source: Nucleic Acids Res. 2015 Jul 21;43(21):e147. doi: 10.1093/nar/gkv733 (PMC4666384; doi:10.1093/nar/gkv733)
Supplement: SUPPLEMENTARY DATA [file supp_gkv733_nar-01688-met-z-2015-File010.docx]

**Supplementary to “BayesPI-BAR: A New Biophysical Model for Characterization of Regulatory Sequence Variations”**

**Junbai Wang and Kirill Batmanov**

**Supplementary Data**

All 67 verified SNPs (1-4) are available in the Excel file.

**Supplementary Figures**

**SFigure 1. Illustration of the principal component projection.** For simplicity, only two dimensions are plotted: δdbA(μ = 0) and δdbA(μ = -10). The real computation uses six dimensions. All data is for a single SNP. Each data point (black circle) represents a PWM, coordinates being its δdbA scores. Data points lie roughly along a line, because the coordinates are strongly correlated. The first principal component of the data set shows the direction of this line, plotted in red. Each data point is projected onto the line, and the coordinate of this projection on the line, a single number, is the combined score. PWMs are ranked according to these projected coordinates. It can be seen how PCA prefers the less noisy δdbA(μ = -10) coordinate to δdbA(μ = 0) by setting the line direction almost horizontal.

**SFigure 2.** **Prediction results of BayesPI-BAR at 47 known regulatory mutations.** **A**) Top left, the heat map of log10-transformed absolute δdbA (the shifted dbA between the reference sequence and the mutated sequence) for 47 known regulatory SNPs (2-4) that affect TF binding to the target gene (*gene name*:TF name). The chemical potentials range between 0 and -23 (the larger the negative value, the higher the protein concentration or chemical potentials). **B**) Top right, the mean and standard deviation of the log10-transformed absolute δdbA values of 47 known *gene*-TF pairs based on different chemical potentials; the red and blue circles represent predictions based on the Maxwell-Boltzmann function and Fermi-Dirac function, respectively. **C**) Bottom left, the regulatory mutations of *gene*-TF pair with the associated TF ranking order, which were predicted for different chemical potentials. **D**) Bottom right, the bar plot of the percentage of TFs with associated ranks for different chemical potentials.


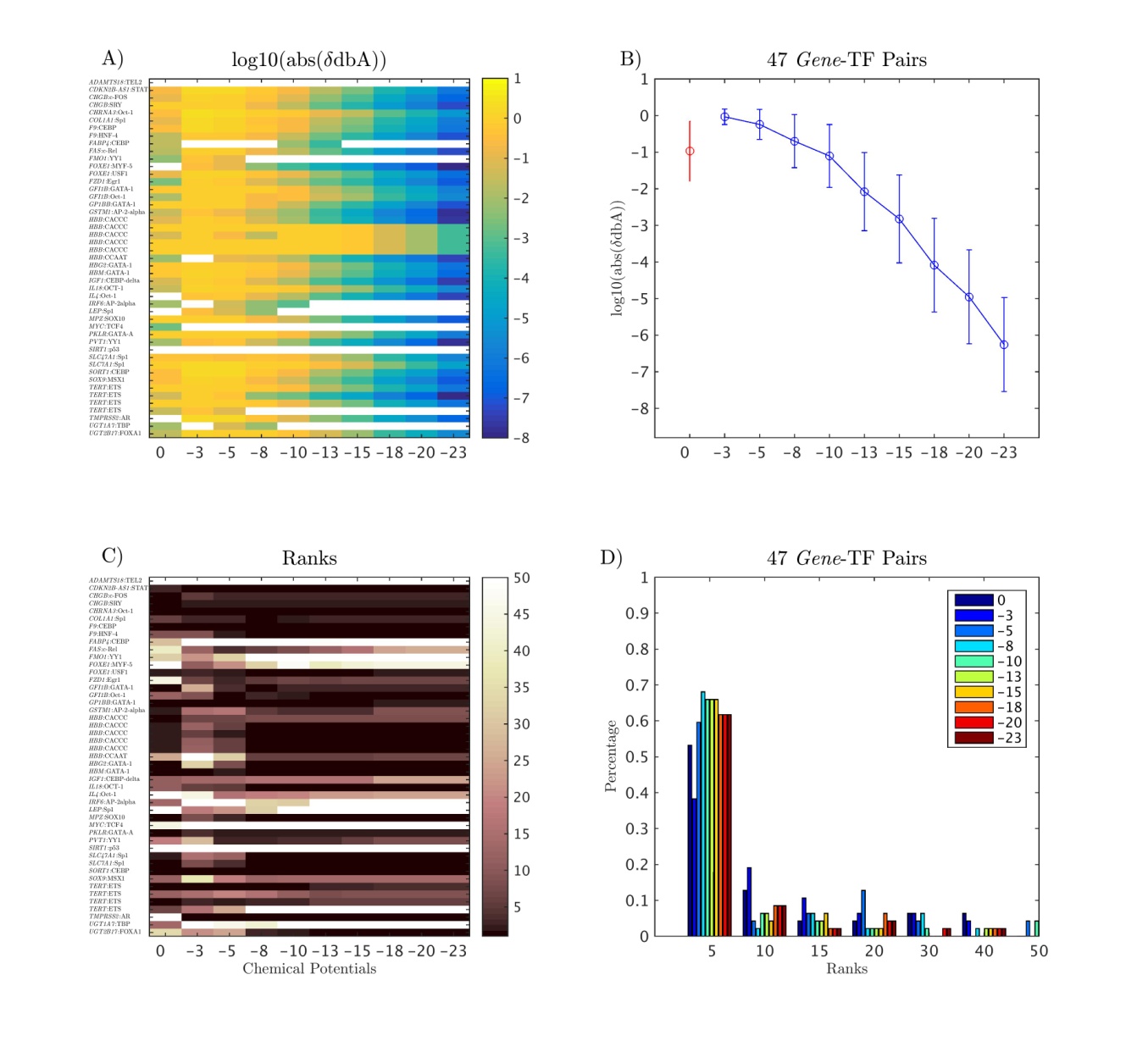


**SFigure 3.** **Prediction results of BayesPI-BAR at 47 known regulatory mutations with different chemical potentials.** **A**) Percentages of SNPs with associated ranks for 47 *gene*-TF pairs (2-4), the predicted TF ranks of known regulatory SNPs are based on chemical potential 0, the median ranks or δdbA for six chemical potentials (0, -10, -13, -15, -18, and -20), and chemical potential -20, respectively. **B**) The same plot as **A)** but for eight chemical potentials (0, -5,-8, -10, -13, -15, -18, and -20).


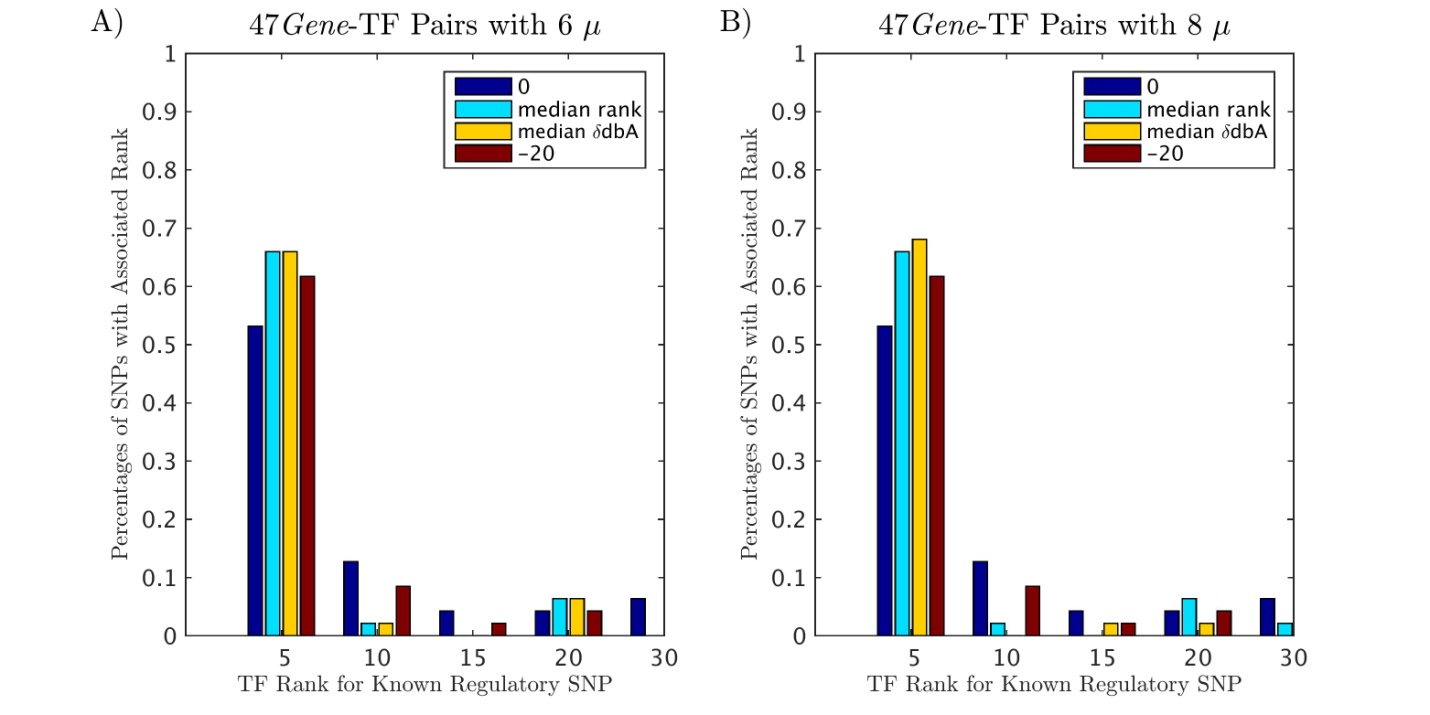


**SFigure 4. Cumulative accuracy of PCA method by using different sequence length.** Cumulative accuracy plots for PCA, the positive/negative direction is not considered, by comparing results based on different length of DNA sequence (i.e. using 61bp and 21bp flanking region of SNPs). It can be seen that the method is not severely affected by the sequence reduction. However, the longer sequences give somewhat better results overall, especially in the lower ranks.


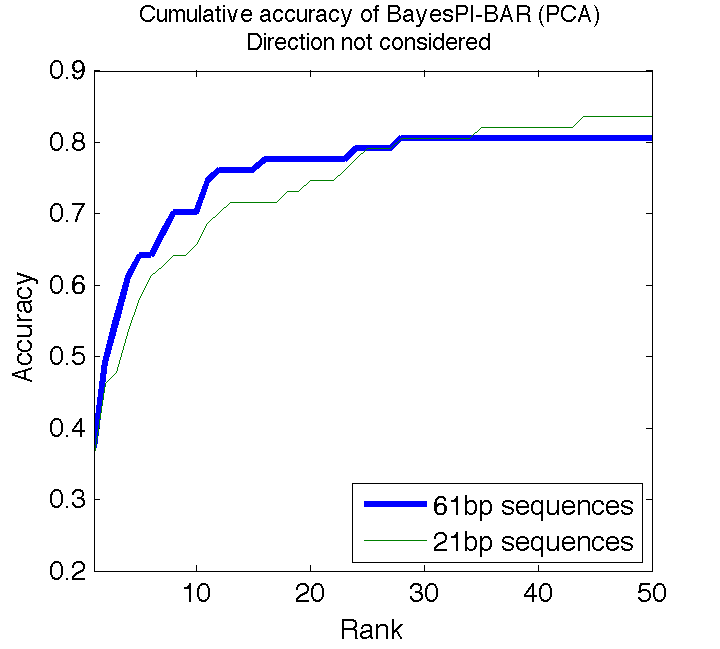


**SFigure 5. Box plot of the top 20 TFs binding affinity changes at regulatory SNPs.** In each row of the figure, “rare,” “Comm,” “HGMD,” and “Rand” represent rare variants, common variants from 1000 genomes (+/-500bp to the TSS), disease associated regulatory SNPs disrupting TF binding sites that predicted by HGMD database (+/-3000bp to the TSS), and randomly generated regulatory SNPs (+/-500bp to the TSS), respectively. Each row of box plots displays the results of the top 20 TFs binding affinity changes (i.e. the sum of positive/negative binding affinity changes, the mean absolute binding affinity changes) were predicted by BayesPI-B from the above-mentioned four categories, respectively.

**
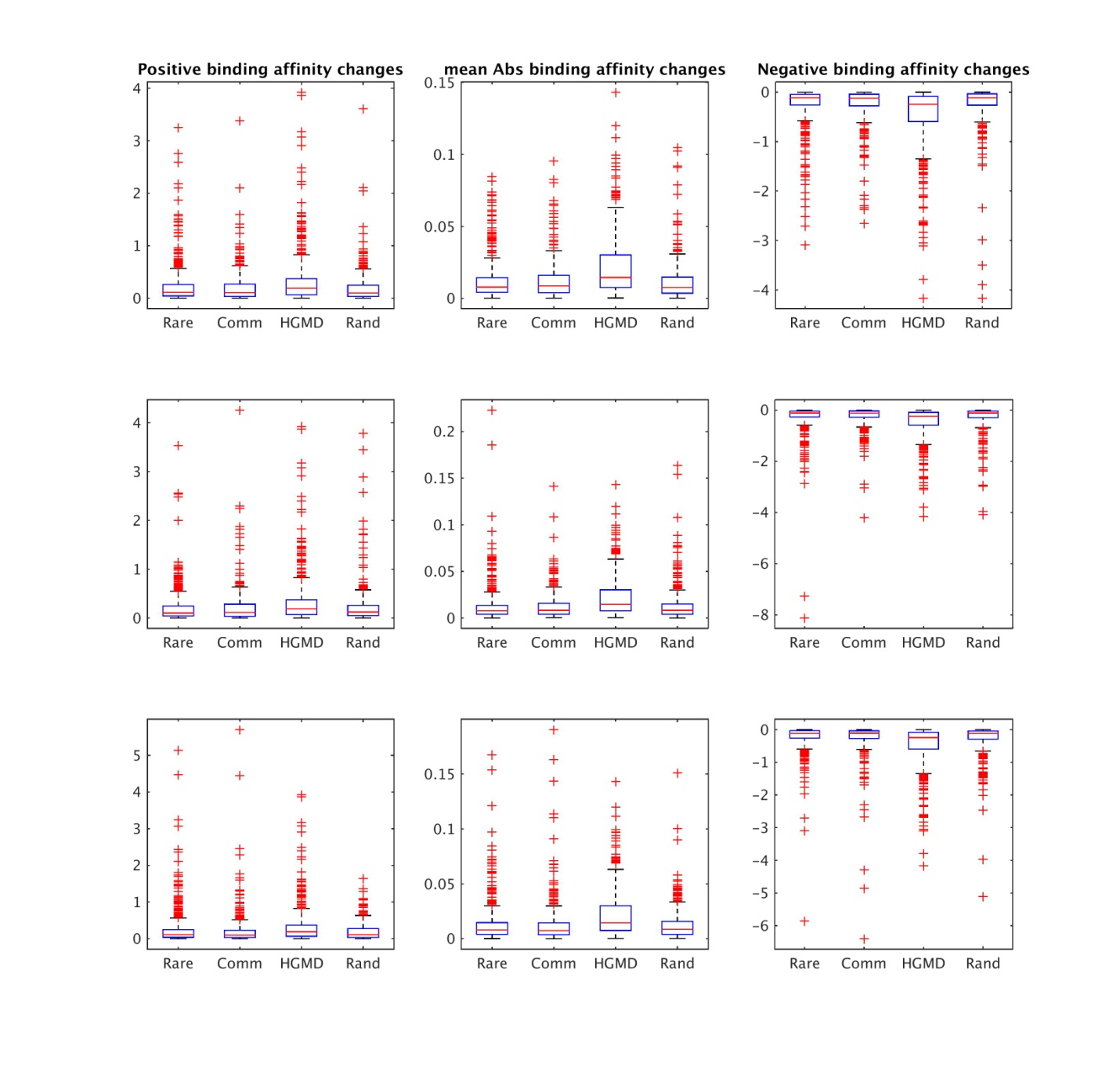
**

**SFigure 6. The distribution of variants at the transcription start site (TSS).** In each row of the figure, “rare,” “Common,” “HGMD,” and “Random” represent rare variants (~141914), common variants (~80561) from 1000 genomes (+/-500bp to the TSS), disease-associated regulatory SNPs disrupting TF binding sites (~416) that predicted by HGMD database (+/-500bp to the TSS), and three times random generation of regulatory SNPs (~1200; +/-500bp to the TSS), respectively. Here, +/-500bp sequences at the TSS are divided into 100 bins, the number of variants in each bin is shown in the lower panel of the figure. At the upper panel of the figure, the distribution of variants is normalized by the maximum number of variants in the bins.


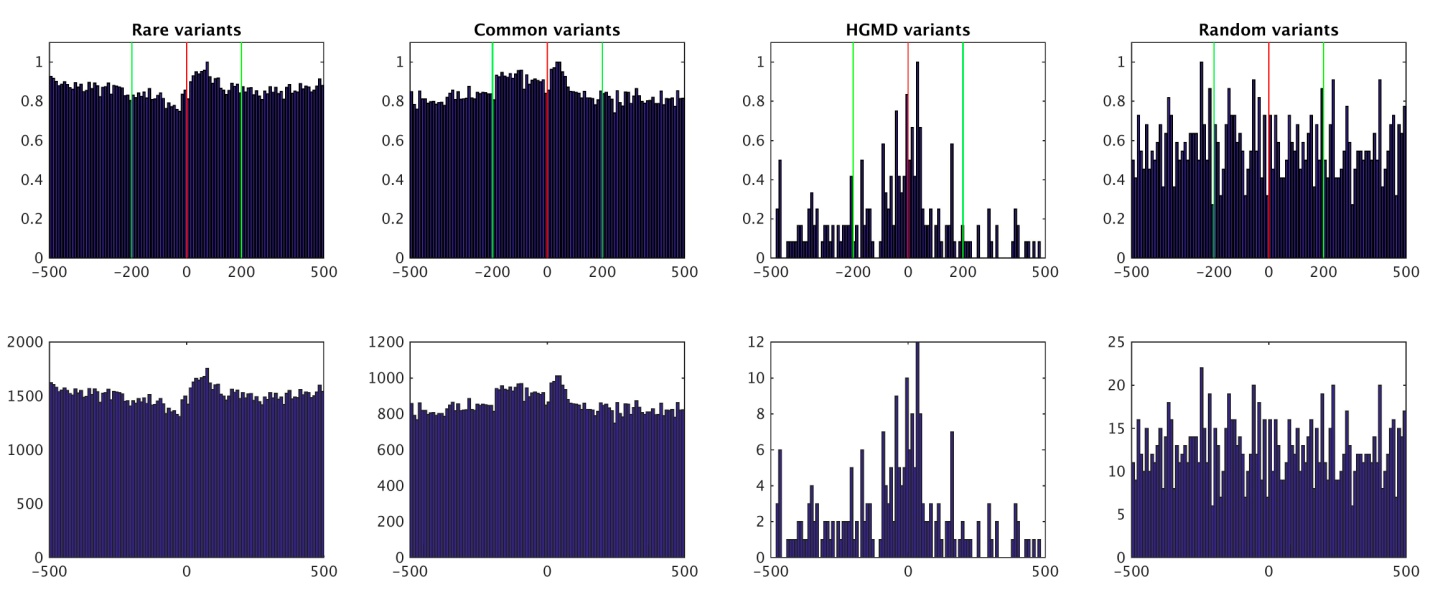


**References**

1. Andersen, M.C., Engstrom, P.G., Lithwick, S., Arenillas, D., Eriksson, P., Lenhard, B., Wasserman, W.W. and Odeberg, J. (2008) In silico detection of sequence variations modifying transcriptional regulation. *PLoS computational biology*, **4**, e5.

2. Epstein, D.J. (2009) Cis-regulatory mutations in human disease. *Briefings in functional genomics & proteomics*, **8**, 310-316.

3. Stenson, P.D., Mort, M., Ball, E.V., Shaw, K., Phillips, A. and Cooper, D.N. The Human Gene Mutation Database: building a comprehensive mutation repository for clinical and molecular genetics, diagnostic testing and personalized genomic medicine. *Human genetics*, **133**, 1-9.

4. Fredriksson, N.J., Ny, L., Nilsson, J.A. and Larsson, E. Systematic analysis of noncoding somatic mutations and gene expression alterations across 14 tumor types. *Nature genetics*, **46**, 1258-1263.
